# Microinjection of Synthetic Peptides Into *Caenorhabditis elegans*

**DOI:** 10.21769/BioProtoc.5640

**Published:** 2026-04-05

**Authors:** Hayao Ohno, Takanori Ida, Yuichi Iino

**Affiliations:** 1 Division of Material and Biological Sciences, Graduate School of Science, Japan Women's University, Tokyo, Japan; 2 Department of Chemical and Biological Sciences, Faculty of Science, Japan Women's University, Tokyo, Japan; 3 Department of Veterinary Physiology, Faculty of Agriculture, University of Miyazaki, Miyazaki, Japan; 4 Center for Animal Disease Control, University of Miyazaki, Miyazaki, Japan; 5 Department of Biological Sciences, School of Science, The University of Tokyo, Tokyo, Japan

**Keywords:** Bioactive peptides, *C. elegans*, In vivo experiment, Microinjection, Chemical synthesis

## Abstract

The genome of the nematode *Caenorhabditis elegans* encodes at least 160 predicted peptide precursor genes that can generate over 300 bioactive peptides, the functions of most of which remain unknown. Phenotypes resulting from deletion or transgenic expression of peptide genes are readily assayed, but genetic dissection of individual peptide activities is often confounded when a single gene encodes multiple peptides or when distinct peptides act redundantly. Here, we describe a protocol for direct microinjection of chemically synthesized peptides into individual worms. This approach permits investigation of the effects of an individual peptide while providing precise temporal control over peptide delivery.

Key features

• Direct injection of chemically synthesized peptides into nematodes.

• In vivo effects can be analyzed within one day after peptide delivery.

• Full control over peptides, chemical modifications, genetic background, and timing of delivery.

• The approach may be adaptable to non-peptide compounds.

## Graphical overview


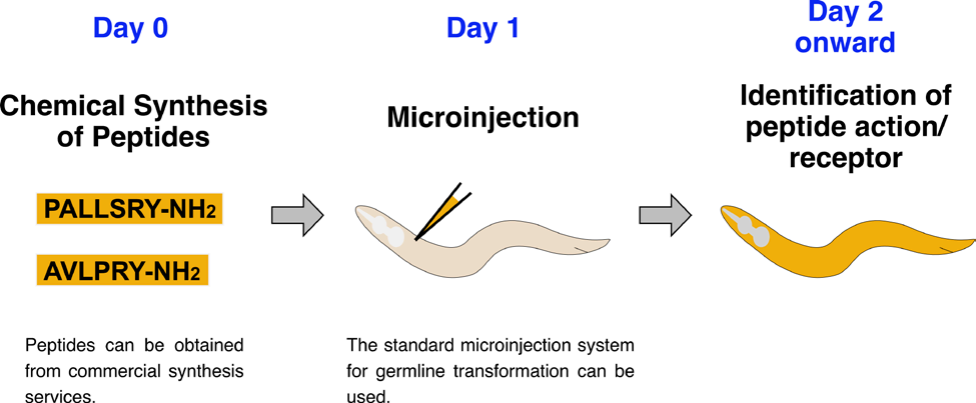


**Direct microinjection of chemically synthesized peptides into *C. elegans*.** To determine the in vivo effects of a specific peptide(s) and to identify receptor mutations that abolish those effects, chemically synthesized peptide(s) are microinjected into worms.

## Background

*Caenorhabditis elegans* has a fully mapped synaptic connectome [1,2], yet understanding of how its nervous system functions remains far from complete, in part because of peptide-mediated signaling between distant cells. Bioinformatic and peptidomic analyses in *C. elegans* have identified 160 peptide-coding genes; post-translational processing of their protein products yields more than 300 distinct peptides [3–5]. Although some of these peptides may be released from non-neuronal cells, the majority are thought to function as neuropeptides [6,7].

Genetic analysis of peptide function in *C. elegans* faces several practical challenges. First, individual peptide genes typically encode multiple distinct peptides; therefore, phenotypes resulting from gene deletion or overexpression do not reveal which peptide(s) are responsible. Second, multiple peptides commonly act on the same receptor [7], such that deletion of a single peptide gene often produces no detectable phenotype. Third, standard genetic manipulations that delete or overexpress peptide genes do not allow precise temporal control of peptide expression.

The protocol we describe here—direct microinjection of chemically synthesized peptides into *C. elegans* [8,9]—addresses these issues; it enables the introduction of individual peptides, may reveal phenotypes that loss-of-function genetics miss, and allows control over the timing of peptide delivery. Furthermore, the approach can be adapted for chemically modified peptides or non-peptidic compounds. However, injected peptide concentrations can differ substantially from endogenous levels and may produce nonphysiological effects, and some peptide actions may require specific combinations of different peptides. Interpreting results is best done in conjunction with complementary approaches [5,10], such as pharmacological characterization and receptor mutant analysis.

## Materials and reagents

**Biological materials**

1. *C. elegans* animals [e.g., available from *Caenorhabditis* Genetics Center (CGC), University of Minnesota, MN, USA]

2. *Escherichia coli* strain used as a food source (e.g., HB101 or OP50, available from the CGC)

**Reagents**

1. Synthetic peptide of interest; peptides can be obtained from commercial synthesis services (e.g., Scrum Inc., Tokyo, Japan)

*Note: As little as 1 nmol is sufficient for the assays.*

2. Trifluoroacetic acid (TFA) (Nacalai Tesque Inc., catalog number: 34840-34)

3. Distilled water (Nacalai Tesque Inc., catalog number: 14029-33)

4. Acetonitrile (Nacalai Tesque Inc., catalog number: 00430-83)

5. Milli-Q water (Millipore Corporation, Milli-Q Advantage A10)

6. Milli-RO water (Millipore Corporation, Elix Essential UV10)

7. Peptone (Gibco Bacto Peptone, catalog number: 211677)

8. Sodium chloride (NaCl) (FUJIFILM Wako Pure Chemical, catalog number: 191-01665)

9. Cholesterol (FUJIFILM Wako Pure Chemical, catalog number: 034-03002)

10. Ethyl alcohol (Ethanol) (Sigma-Aldrich, catalog number: 09-0770)

11. Agar (TAISHO TECHNOS, catalog number: 17071902)

12. Calcium chloride dihydrate (CaCl_2_·2H_2_O) (FUJIFILM Wako Pure Chemical, catalog number: 033-25035)

13. Magnesium sulfate heptahydrate (MgSO_4_·7H_2_O) (FUJIFILM Wako Pure Chemical, catalog number: 131-00405)

14. Potassium dihydrogen phosphate (KH_2_PO_4_) (FUJIFILM Wako Pure Chemical, catalog number: 169-04245)

15. Disodium hydrogen phosphate dodecahydrate (Na_2_HPO_4_·12H_2_O) (FUJIFILM Wako Pure Chemical, catalog number: 196-02835)

16. Dipotassium hydrogen phosphate (K_2_HPO_4_) (FUJIFILM Wako Pure Chemical, catalog number: 164-04295)

17. Potassium hydroxide (KOH) (FUJIFILM Wako Pure Chemical, catalog number: 168-21815)

18. Agarose (Nacalai Tesque Inc., catalog number: 01157-95)

19. Luria-Bertani (LB) broth (BD Biosciences, catalog number: 244620)

20. Liquid paraffin (FUJIFILM Wako Pure Chemical, catalog number: 128-04375) or Halocarbon oil 700 (Sigma-Aldrich, catalog number: H8898-100ML)

**Solutions**

1. 10% (v/v) TFA (stock) (see Recipes)

2. 0.1% TFA (see Recipes)

3. HPLC solvent B (see Recipes)

4. LB liquid medium (see Recipes)

5. 5 mg/mL cholesterol (see Recipes)

6. 1 M potassium phosphate buffer (pH 6.0) stock solution (see Recipes)

7. M9 buffer (see Recipes)

8. Nematode growth media (NGM) plates (see Recipes)

**Recipes**

**1. 10% (v/v) TFA (stock)**

**Table:** 

Reagent	Final concentration	Quantity or volume
100% TFA	10%	1 mL
Distilled water	n/a	Up to 10 mL
Total		10 mL

Store at 4 °C in an amber glass bottle; it is functional for at least 1 year.

**2. 0.1% TFA**

**Table:** 

Reagent	Final concentration	Quantity or volume
10% TFA	0.1%	1 mL
Distilled water	n/a	Up to 100 mL
Total		100 mL

Prepare 0.1% TFA by diluting the 10% stock. Store at room temperature in an amber glass bottle and use within 1 year.

**3. HPLC solvent B**

**Table:** 

Reagent	Final concentration	Quantity or volume
Acetonitrile	40%	40 mL
10% TFA	0.1%	1 mL
Distilled water	n/a	Up to 100 mL
Total		100 mL

Store at room temperature in an amber glass bottle and use within 1 year. To enhance reproducibility, use graduated cylinders and glass pipettes reserved exclusively for this solution when measuring volumes.

**4. LB liquid medium**

**Table:** 

Reagent	Final concentration	Quantity or volume
LB broth	25 g/L	2.5 g
Milli-RO water	n/a	Up to 100 mL
Total		100 mL

Autoclave for 20 min in a 300–500 mL flask. Store at room temperature and use within 2 months.

**5. 5 mg/mL cholesterol**

**Table:** 

Reagent	Final concentration	Quantity or volume
Cholesterol	5 mg/mL	0.5 g
Ethanol	n/a	100 mL

Leave the suspension in a sterilized bottle overnight to ensure complete dissolution, mix the solution thoroughly, store at room temperature, and use within 1 year.

**6. 1 M potassium phosphate buffer (pH 6.0) stock solution**

**Table:** 

Reagent	Final concentration	Quantity or volume
KH_2_PO_4_	1 M	136.1 g
KOH	n/a	As required to adjust pH
Milli-Q water	n/a	Up to 1 L
Total		1 L

Dissolve in ~800 mL of Milli-Q water, add KOH pellets to pH 6.0, and bring the volume to 1 L with Milli-Q water. Autoclave for 20 min. Store at room temperature; it is functional for at least 1 year.

**7. M9 buffer**

**Table:** 

Reagent	Final concentration	Quantity or volume
KH_2_PO_4_	3 g/L	0.3 g
Na_2_HPO_4_·12H_2_O	15.2 g/L	1.52 g
NaCl	5 g/L	0.5 g
Milli-Q water	n/a	Up to 100 mL
Total		100 mL

Sterilize by autoclaving for 20 min at 121 °C and add 100 μL of 1 M MgSO_4_ after cooling to room temperature (final concentration: 1 mM). Store at room temperature; it is functional for at least 1 year.

**8. NGM plates**

**Table:** 

Reagent	Final concentration	Quantity or volume
Peptone	2.5 g/L	2.5 g
NaCl	3 g/L	3 g
Agar	17 g/L	17 g
5 mg/mL cholesterol	5 mg/L	1 mL
Milli-RO water	n/a	800 mL

Autoclave for 30 min in a 1 L Erlenmeyer flask. Let the NGM cool to ~65 °C. Add 100 mL of 10 mM CaCl_2_, 100 mL of 10 mM MgSO_4_, and 25 mL of 1 M potassium phosphate buffer (pH 6.0) and thoroughly mix the medium. Dispense 10 mL per 60 × 15 mm Petri dish. Leave the plates at room temperature for ~48 h under low-humidity conditions or ~72 h under high-humidity conditions; then, use them immediately or store at 4 °C and use within 2 months.

**Laboratory supplies**

1. Sterile pipette tips

2. 50 mL conical polypropylene centrifuge tubes

3. 0.5 mL PCR single tube (SARSTEDT, catalog number: 72.735.002)

4. Glass capillary with filament (NARISHIGE, catalog number: GDC-1)

5. Capillary loading tips (Eppendorf, catalog number: 5242956003)

6. Cover glass, 24 mm × 50 mm (Matsunami Glass, catalog number: C018181)

7. Cover glass, 18 mm × 18 mm (Matsunami Glass, catalog number: C024501)

8. 1.5 mL screw-cap microtubes (SARSTEDT, catalog number: 72.692J)

9. Petri dishes, 60 mm × 15 mm (Greiner Bio-One, catalog number: 628161)

10. Platinum wire pick to transfer *C. elegans*

11. 0.22 μm filter column (Merck Millipore, catalog number: SLGV004SL)

## Equipment

**Figure 1.**
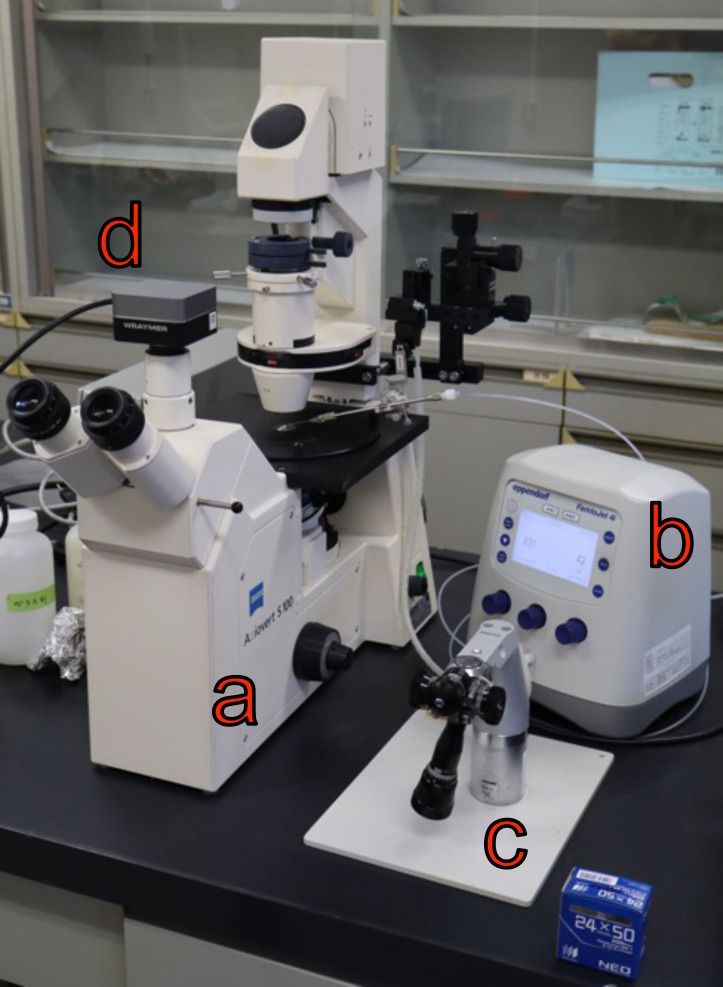
Example of a microinjection system setup. (a) Inverted microscope Axiovert S 100; (b) microinjector FemtoJet 4i; (c) micromanipulator MMO-4; (d) camera WRAYCAM-VEX832.

1. Vacuum oil pump (Thermo Scientific, model: VLP200)

2. Refrigerated vapor trap (Thermo Scientific, model: RVT4104)

3. Desiccator (Sankyo, catalog number: 85-5131)

4. Diaphragm-type dry vacuum pump (ULVAC, model: DAP-15)

5. Microcentrifuge (e.g., TOMY, model: MC-150)

6. -80 °C freezer

7. Microwave

8. Stereomicroscope

9. Glass micropipette puller (NARISHIGE, model: PB-7)

10. Inverted microscope for microinjection [ZEISS, model: Axiovert S 100 equipped with differential interference contrast (DIC) prisms, gliding table, objective 40×/0.75 EC Plan-NEOFLUAR] (Figure 1)

11. Microinjector unit (e.g., Eppendorf, model: FemtoJet 4i) (Figure 1)

12. Micromanipulator (NARISHIGE, model: MMO-4) (Figure 1)

13. (optional) Microscope camera (Wraymer, model: WRAYCAM-VEX832) (Figure 1)

## Software and datasets

1. MicroStudio (Wraymer, Version 1.9.25633.20240519) for movie acquisition from WRAYCAM-VEX832 (optional)

2. ImageJ/Fiji (NIH, Version 2) for image and video analysis (optional)

## Procedure

**Overview and timeline**

The protocol consists of five main phases:

A. Preparation of injection pads and injection needles (~1 h).

B. Preparation of peptides (~1–2 days).

C. Preparation of worms (~1 week).

D. Microinjection (~2–6 h).

E. Phenotype analysis (several hours to several days; several weeks if measuring lifespan)

**A. Preparation of injection pads and injection needles**

1. Boil ~20 mL of 2% (w/v in water) agarose in a 100 mL glass bottle by heating in a microwave.

2. Place ~10 μL of the molten agarose onto the center of a 24 × 50 mm coverslip, then immediately place a second 24 × 50 mm coverslip on top to flatten the drop.

*Note: Lowering the temperature of the agarose solution during pad formation increases the thickness of the resulting agarose pads. Although thicker pads facilitate immobilization of worms, they also exacerbate dehydration-induced damage. We typically spread ~10 μL of the 2% agarose solution into a circular pad about 1.5 cm in diameter; however, you should determine the optimal pad thickness empirically.*

3. Separate the coverslips and dry the pad in a 60 °C incubator for at least several hours. The injection pads can be stored in bulk at room temperature.

**Figure 2.**
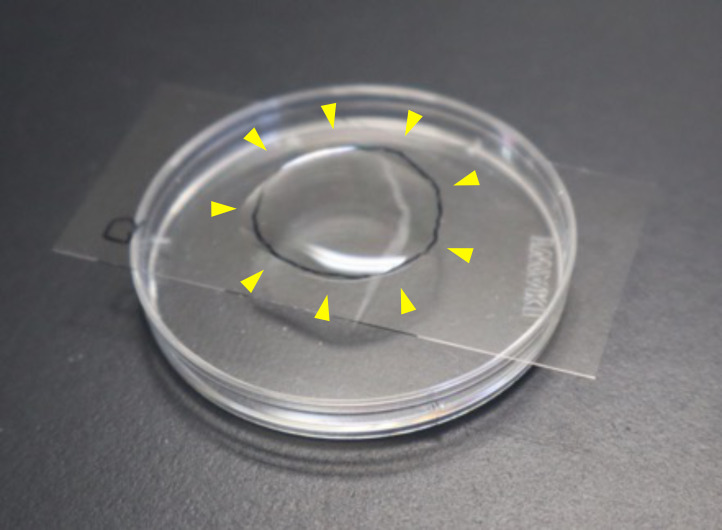
An injection pad. A droplet of liquid paraffin (described in section D) was placed on the injection pad (circled, yellow arrowheads).

*Note: Tracing the outline of the agarose on the back side of the coverslip with an oil-based marker makes the pad location easy to find. Marking one corner of the coverslip (for example, the lower-left corner) with the same marker and maintaining a consistent orientation prevents confusion between the front and back (Figure 2).*

4. Mount a GDC-1 glass capillary in a micropipette puller and pull it at 65–70 °C to produce two injection needles.

*Note: Ensure that the tip thickness and the size of its opening are appropriate. Typically, needles with a tip opening diameter of ~1 μm and a conical taper length of about 5 mm are used. Tips that are too fine clog easily, whereas tips that are too thick do not penetrate tissue reliably. If the tip opening is too small to allow fluid flow, widen it as follows: place an 18 × 18 mm coverslip on a 24 × 50 mm coverslip, drop ~50 μL of liquid paraffin at the edge of the smaller coverslip, bring the needle tip into contact with that edge using a micromanipulator under a microscope, and gently tap the microscope stage or the needle holder. Alternatively, pre-pulled needles are commercially available (Eppendorf, Femtotips II, catalog number: 930000043 or Tritech Research, MINJ-PP).*

**B. Preparation of peptides**

1. Transfer 30 mL or 0.1% TFA to a 50 mL conical centrifuge tube.

*Note: To remove coating residues and other substances, pre-wash the 50 mL conical tube by adding approximately 5 mL of 0.1% TFA, vortexing, discarding the liquid, and repeating this wash three times.*

2. Add 500 μL of HPLC solvent B to each of two 1.5 mL screw-cap microtubes and vortex. Centrifuge the tube at 1,000× *g* for a few seconds at room temperature, open the caps, and remove the liquid by aspirating with a pipette tip connected to a diaphragm-type dry vacuum pump.

3. Repeat step B2 two additional times (total of three washes). Allow the tubes to dry before use.

4. Dissolve a lyophilized synthetic peptide of interest, which can be obtained from commercial synthesis services, in 0.1% TFA to a final concentration of 10^-2^ M.

*Note: For pipetting 0.1% TFA or the peptide solution, precondition each pipette tip by aspirating and discarding 0.1% TFA three times before use to remove coating residues and other substances that could leach from its inner surface.*

5. Transfer 10 μL (100 nmol) of the 10^-2^ M peptide solution into a 1.5 mL screw-cap microtube washed with HPLC solvent B in steps B2–3 and lyophilize as follows:

a. Close the cap, spin briefly, then loosen the cap.

b. Freeze at -80 °C for 1 h.

c. Place the tube in a desiccator and dry overnight with a vacuum oil pump connected with a refrigerated vapor trap.

d. Remove the tube from the desiccator and tighten the cap.

**Pause point:** Lyophilized peptides can be stored at -20 °C for at least 1 year.

6. Redissolve the lyophilized peptide in 1 mL of 0.1% TFA to a 10^-4^ M solution.

7. Transfer 10 μL (1 nmol) of the 10^−4^ M peptide solution into a 1.5 mL screw-cap microtube washed with HPLC solvent B in steps B2–3, and lyophilize by repeating step B5a–d.

**Pause point:** Lyophilized peptides can be stored at -20 °C for at least 1 year.

**C. Preparation of worms**

1. Inoculate *Escherichia coli* into LB liquid medium and incubate with shaking at 37 °C overnight.

2. Pipette 200 μL of the overnight culture onto the center of each NGM plate (see Recipes), incubate the plates at room temperature for ~48 h, and then use them immediately or store them at 4 °C and use within 2 months.

3. Grow *C. elegans* worms on the *E. coli*–seeded NGM plates to the desired developmental stage [11]. For example, transfer L4 larvae at the white crescent stage to fresh plates using a stereomicroscope and a platinum wire pick and incubate at 20 °C for 36 h to obtain 1-day-old adult animals.

**D. Microinjection**

1. Dissolve the lyophilized peptide (1 nmol, see above) in 100 μL of M9 buffer to give a 10^-5^ M solution.

*Note: Peptides can adsorb to tube walls over time after dissolution, reducing activity. Use the solution immediately or store the peptide in the lyophilized state until use.*

**Figure 3.**
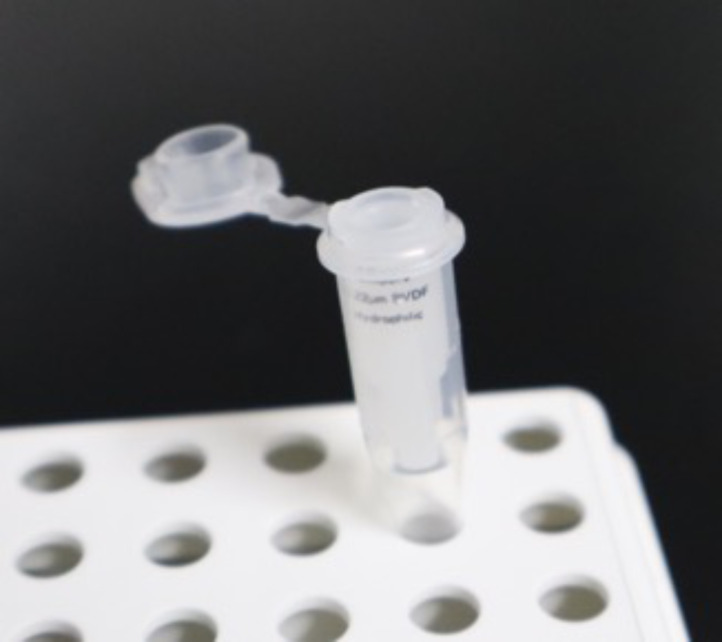
Filter column set in a 0.5 mL tube

2. Insert a 0.22 μm filter column into a 0.5 mL PCR single tube (Figure 3), load 10 μL of the peptide solution onto the filter, and centrifuge the tube at 5,000× *g* for 1 min.

3. Remove the filter column and centrifuge the filtrate in a microcentrifuge at maximum speed for 10 min to pellet particulates to reduce the risk of capillary clogging.

4. Wipe the area around an inverted microscope for microinjection with 70% ethanol to prevent contamination.

5. Place ~100 μL of liquid paraffin onto an injection pad (Figure 2).

6. Turn on the microinjection unit and set the injection pressures to Pi = 500 and Pc = 20. If the unit allows setting the injection volume, set it to 100 fL.

7. Load ~1 μL of the peptide solution (step D3) into an injection needle (see above) using a capillary loading tip.

8. Hold the needle vertically for ~1 min to allow bubbles to rise and escape. Confirm under a stereomicroscope that no bubbles are present at the tip.

9. Mount the needle into a needle holder attached to a micromanipulator.

10. Confirm that the micromanipulator retains sufficient travel range in all directions.

11. Position the micromanipulator so that the needle tip lies at the center of the field of view of the inverted microscope.

12. Raise the needle tip upward with the micromanipulator to make space for placement of the injection pad.

13. Transfer a worm at the appropriate developmental stage (see above) to a fresh, *E. coli*-free NGM plate and let the worm move around for ~20–30 s to remove adherent bacteria; eliminating bacterial carryover increases injection efficiency. Pick the worm onto the injection pad and wait until it is immobilized.

14. Place the injection pad on the stage of the inverted microscope and center the worm in the field of view.

15. Lower the needle tip using a micromanipulator and adjust focus so that both the pharyngeal region and the needle tip are sharply in view.

16. Advance the needle into the head in a direction from the midbody toward the head tip (Video 1). Inject the solution slightly posterior to the dorsal part of the pharyngeal terminal bulb until the spread diameter reaches approximately 5–10 μm (≈1/6 to 1/3 of the terminal-bulb diameter). The injected volume is roughly estimated to be around 100 fL, depending on the degree of vertical compression of the worm.

*Note: As a control, inject peptide-free M9 buffer (vehicle) or an unrelated peptide. The injection site need not be restricted to the head; select a location that is not far from the putative receptor cells and unlikely to influence the phenotype of interest.*

17. While viewing under a stereomicroscope, apply 1 μL of M9 buffer to the worm and confirm detachment and movement.

**E. Phenotype analysis**

1. Transfer injected worms to *E. coli*–seeded NGM plates using a stereomicroscope and a platinum wire pick.

2. After incubation at 20–23 °C for an appropriate interval, assess the phenotype of interest. The latency to detectable peptide effects depends on the peptide and the phenotype; for example, LURY-1-mediated modulation of pharyngeal pumping and egg-laying behavior is apparent 3–4 h after microinjection [8].

**Video 1.**
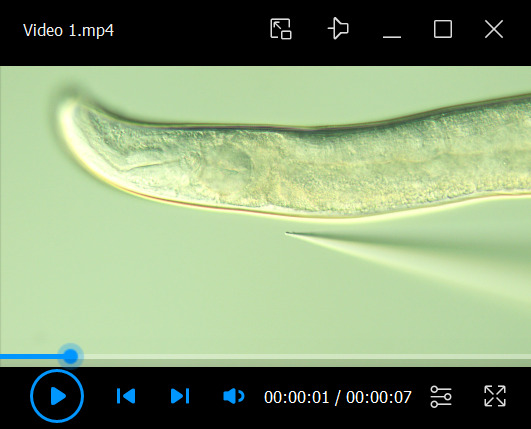
Microinjection. This video shows the injection of a peptide-containing solution into the head region of a worm. To facilitate visualization, the injection solution was stained with bromophenol blue, and a slightly larger volume than used in our standard protocol was delivered.

## Validation of protocol

This protocol has been used and validated in the following research article:

• Ohno et al. [8]. Luqin-like RYamide peptides regulate food-evoked responses in *C. elegans. eLife* (Figure 5A, B and Figure 5, figure supplement 1C–D).

Raw data that support the findings of the article are available at Ohno-2017-elife/5362588/2">https://figshare.com/articles/dataset/Ohno-2017-elife/5362588/2.

## General notes and troubleshooting

**General notes**

1. The feasibility of applying this method to large-scale screening depends on proficiency with the injection technique. With sufficient practice, a skilled experimenter can inject hundreds of worms per day. For screening many candidate peptides, one feasible strategy would be to pool peptides into groups (e.g., several to a few dozen peptides per pool), inject each pool, and identify which pools contain the active peptide(s).

2. Injected peptides do not necessarily mimic the concentration or temporal pattern of endogenous peptide expression. Therefore, the concentration and timing of peptide delivery and the interpretive validity of any resulting phenotypes should be evaluated carefully. Peptide purity is also an important factor when determining the concentration for administration; peptides should be produced at the highest practicable purity.

3. Variations in injected volume and injection-induced tissue damage can contribute to phenotype variability. The required sample size depends on the strength and type of phenotype being observed; in some cases, it may be larger than that required for genetic mutant analyses or other approaches.
